# Does antithrombotic therapy improve survival with colorectal cancer?

**DOI:** 10.1186/s12957-017-1235-z

**Published:** 2017-08-24

**Authors:** Kodai Takahashi, Hideto Ito, Masatoshi Hashimoto, Kazuhito Mita, Hideki Asakawa, Takashi Hayashi, Keiichi Fujino

**Affiliations:** Department of Surgery, New-Tokyo Hospital, 1271 Wanagaya, Matsudo-city, Chiba 270-2232 Japan

**Keywords:** Colorectal cancer, Antithrombotic therapy, Antiplatelet therapy

## Abstract

**Background:**

The study aimed to evaluate the prognosis for patients with colorectal cancer who underwent surgery while receiving antithrombotic therapy (ATT) across all disease stages and for patients at disease stages 0–III.

**Methods:**

This retrospective cohort study included 710 Japanese patients who underwent surgery for colorectal cancer between January 2009 and November 2015 at our institution. Approximately 35% of these patients received ATT. Of these, 199 (28.0%) received antiplatelet therapy, and 76 (10.7%) received anticoagulant therapy. We investigated the prognosis among patients with colorectal cancer receiving ATT, antiplatelet therapy, or anticoagulant therapy in all-stage and stage 0–III cancers.

**Results:**

For all disease stages combined, no benefit was observed for ATT, antiplatelet therapy, and anticoagulant therapy groups in the overall survival rates (ATT: 87.8 vs. 78.4%, *P* = 0.23; antiplatelet therapy: 87.8 vs. 78.6%, *P* = 0.25; and anticoagulant therapy: 92.2 vs. 80.2%, *P* = 0.26). However, overall survival rates of patients with stage 0–III colorectal cancer undergoing ATT, antiplatelet therapy, and anticoagulant therapy significantly improved. (ATT: 98.5 vs. 92.7%, *P* = 0.01; antiplatelet therapy: 98.3 vs. 91.1%, *P* = 0.02; and anticoagulant therapy: 100 vs. 92.1%, *P* = 0.00).

**Conclusion:**

Receiving ATT significantly improves overall survival rates in patients with stage 0–III colorectal cancer.

## Background

The incidence of cancer has been increasing in the world due to the increase in average life expectancy. Colorectal cancer is one of the most common malignancies in the world [1, 2]. Previous studies have supported the protective effect of aspirin in reducing overall colorectal cancer incidence and mortality [3–5]. The anti-cancer effects of aspirin were first identified in animal models in 1972 [6], although the mechanism remains unclear in humans [7]. Aspirin has more than one action in its effects on disease. In general, we believe that aspirin’s inhibition of cyclooxygenase (COX) enzymes may mediate some of the effects [8]. According to some studies, other mechanisms may include the reduced flux of L-ornithine through ornithine decarboxylase, which may participate in the antiproliferative activity of aspirin toward colonic tumor cells [9] or blocking of the inflammatory response at the gene level [10] by reducing apoptosis through the release of mitochondrial cytochromes [11] or via the upregulation of Bcl-2 and Bax and suppression of vascular endothelial growth factor [12].

Recent studies have suggested that aspirin reduces the risk of cancer recurrence and improves survival [13, 14]. However, TRITON-TIMI 38 trial showed that dual antiplatelet therapy may result in an increased incidence of solid cancers [15]. It is unclear where anticoagulant and antiplatelet therapy may inhibit tumor development. The relationship between antithrombotic therapy (ATT) and prognosis in patients with colorectal cancer has not yet been reported. We hypothesized that the effect of ATT results in improved survival in patients with colorectal cancer. The aim of the study was to evaluate the prognosis for patients with colorectal cancer who underwent surgery while receiving ATT, both across all disease stages combined and in just patients with stage 0–III disease. In addition, we estimated the prognosis among patients with all-stage and disease stage 0–III colorectal cancer receiving ATT, antiplatelet therapy, or anticoagulant therapy. To the best of our knowledge, this is the first report to specifically focus on prognosis among patients with colorectal cancer who underwent surgery while receiving ATT.

## Methods

All patients provided written informed consent. The study was approved by the Institutional Review Board to ensure the protection of patient privacy and confidentiality. The study was undertaken in accordance with the ethical standards of the World Medical Association Declaration of Helsinki.

This retrospective cohort study included 710 Japanese patients (459 females and 251 males; median age: 70.4 ± 10.6 years) who underwent surgery between January 2009 and November 2015 at our institution. Of the 710 patients, 104 (14.6%) were pathologically diagnosed with stage IV disease and 246 (34.6%) received ATT. All patients undergoing curative resection for stage 0–III colorectal cancer were included. The procedures were performed according to the current guidelines. The stage of disease was evaluated by computed tomography of the thorax, abdomen, and pelvis, and positron emission tomography was performed if clinically indicated. Clinical and pathological staging of the disease was assessed according to the American Joint Committee on Cancer Staging Manual, seventh edition [16]. All pathology specimens from the initial endoscopic biopsies were read and confirmed by pathologists specializing in gastrointestinal malignancies. Pathological examinations included tumor detection and the assessment of invasion depth, the number of metastatic lymph nodes, and surgical margins.

Patient characteristics, the type of surgery, the type of ATT, clinicopathological findings, and prognosis were obtained through a standardized review of the surgery database at our institution. The patients were divided into two groups: ATT users (the ATT group), those who used antithrombotic agents for >3 months continuously prior to the colorectal cancer diagnosis, and non-users (the control group), those who did not use antithrombotic agents. The ATT group was further divided into two subgroups according to whether the patients received antiplatelet or anticoagulant therapy. The indication for the administration of antiplatelet or anticoagulant agents was as follows: patients who underwent percutaneous coronary intervention or coronary artery bypass graft surgery received single or dual antiplatelet therapy (low-dose aspirin 100 mg/day and clopidogrel 75 mg/day) and those with atrial fibrillation, mechanical heart valve, or history of deep vein thrombosis received warfarin or a novel oral anticoagulant (edoxaban 30 mg/day, rivaroxaban 15 mg/day, apixaban 10 mg/day, and dabigatran 300 mg/day).

The analyzed study end-points were overall survival (OS) for patients receiving ATT, antiplatelet therapy, or anticoagulant therapy in those with all-stage disease and in those with stage 0–III. OS was calculated from the date of operation for colorectal cancer to the date of death from any cause. Follow-up data were obtained from the patients’ medical records and their referring physicians. All patients were assessed every 3 months for the first 5 years after the completion of treatment. Routine follow-up examinations included physical examination, a history, and CT scans of the chest/abdomen. Endoscopy was performed if clinically indicated. CT scans of the chest/abdomen were routinely performed every 6 months.

### Statistical analysis

Statistical analysis was performed using JMP® 11 (SAS Institute Inc., Cary, NC, USA). The results are expressed as means ± standard deviation and percentages. The patient characteristics, type of surgery, type of ATT, and clinicopathological findings of the ATT and control groups were compared by Student’s *t* test for continuous variables and the chi-square test or Fisher’s exact test for categorical variables. The patients were followed-up periodically until the final follow-up or death. Differences in the cumulative survival rates between the ATT, antiplatelet therapy, and anticoagulant therapy groups and the control group were calculated by the log-rank test on univariate analysis for comparison using Kaplan–Meier survival curves. A probability (*p*) value of <0.05 was considered statistically significant.

## Results

Around 35% of the patients who underwent surgery for colorectal cancer in our institution received ATT. Of these patients, 199 (28.0%) received antiplatelet therapy, 172 (24.2%) received aspirin, and 76 (10.7%) received anticoagulant therapy. In our cohort, angina pectoris was the major indication for antiplatelet therapy, and atrial fibrillation was the major indication for anticoagulant therapy. Table 1 summarizes the clinicopathological findings in the ATT and control groups.

**Table 1:** Patient characteristics

Characteristic	Overall, *n* = 710	Antithrombotic therapy, *n* = 246	Control, *n* = 464	*p* value
Age (years, mean ± SD)		73.8 ± 7.8	68.6 ± 11.6	0.01
Gender (*n*, %)	Male	183(74.3)	276 (59.4)	
	Female	63 (25.7)	188 (40.6)	0.01
BMI (kg/m2, mean ± SD)		22.3 ± 3.4	23.6 ± 6.7	0.57
ASA score (*n*, %)	1	0 (0)	63 (13.5)	
	2	0 (0)	366 (78.9)	
	3	225 (91.5)	22 (5.0)	
	4	21 (8.5)	13 (2.6)	0.01
Comorbidities (*n*, %)	≤1	18 (7.3)	98 (21.1)	
	>1	228 (92.7)	366 (78.9)	0.02
Tumor location (*n*, %)	Right side	103 (41.5)	209 (45.0)	
	Left side	67 (27.6)	102 (22.0)	
	Rectum	76 (30.9)	153 (33.0)	0.37
Extended resection (*n*, %)	Yes	22 (8.9)	63 (13.5)	
	No	224 (91.1)	401 (86.5)	0.07
Laproscopic surgery (*n*, %)	Yes	52 (21.1)	121 (26.0)	
	No	194 (78.9)	343 (74.0)	0.14
Chemotherapy (*n*, %)	Yes	120 (48.7)	268 (57.7)	
	No	126 (51.3)	196 (42.3)	0.02
Recurrence (*n*, %)	Yes	36 (14.6)	98 (21.1)	
	No	210 (85.4)	366 (78.9)	0.03
*T* stage (*n*, %)	0	9 (3.6)	11 (2.3)	
	1	50 (20.3)	82 (17.6)	
	2	34 (13.8)	44 (9.5)	
	3	100 (40.6)	198 (42.7)	
	4	53 (21.7)	128 (27.9)	0.15
*N* stage (*n*, %)	0	146 (59.3)	281 (60.5)	
	1	60 (24.3)	103 (22.2)	
	2	33 (13.4)	63 (13.5)	
	3	7 (3.0)	16 (3.8)	0.9
*M* factor (*n*, %)	0	220 (89.4)	396 (85.3)	
	1	26 (10.6)	67 (14.7)	0.14
The number of lymph node metastasis (*n*, %)		1.59 ± 3.15	1.47 ± 2.81	0.6
Lymph node metastasis (*n*, %)	Yes	103 (41.8)	179 (38.5)	
	No	143 (58.2)	285 (61.5)	0.39
Stage (*n*, %)	0	9 (3.6)	11 (2.4)	
	I	65 (26.4)	114 (24.6)	
	II	65 (26.4)	142 (30.6)	
	IIIa	54 (21.9)	87 (18.7)	
	IIIb	21 (8.5)	37 (8.0)	
	IV	32 (13.2)	72 (15.7)	0.59
ly (*n*, %)	0	67 (27.2)	106 (22.8)	
	1	140 (56.9)	252 (54.3)	
	2	33 (13.4)	89 (19.2)	
	3	6 (2.5)	16 (3.7)	0.15
v (*n*, %)	0	116 (47.2)	202 (43.5)	
	1	62 (25.2)	118 (25.4)	
	2	61 (24.8)	121 (26.1)	
	3	7 (2.8)	22 (5.0)	0.55
R (*n*, %)	0	222 (90.2)	399 (86.0)	
	1	24 (9.8)	64 (14.0)	0.22
Histrogical type (*n*, %)	Well	212 (86.2)	398 (84.3)	
	Other	34 (13.8)	66 (15.7)	0.88
Poorly differentiated adenocarcinoma (*n*, %)	Yes	18 (7.3)	43 (9.2)	
	No	228 (92.7)	421 (90.8)	0.37
Mucinous adenocarcinoma (*n*, %)	Yes	19 (7.7)	31 (6.7)	
	No	227 (92.3)	433 (93.3)	0.61

There were no significant differences between the control and ATT groups in the rate of extended resection or the rate of laparoscopic surgery. The mean age was significantly higher in the ATT group than that in the control group (73.8 ± 7.8 vs. 68.6 ± 11.6, *P* = 0.01). The proportion of males in the ATT group was significantly higher than that in the control group (74.3 vs. 59.4%, *P* = 0.01, univariate analysis). The rate of chemotherapy and the rate of recurrence was significantly greater in the control group than that in the ATT group (48.7 vs. 57.7%, *P* = 0.02, univariate analysis and 14.6 vs. 21.1%, *P* = 0.03, univariate analysis, respectively). With regard to pathological findings, there were no significant differences between the control and ATT groups in *T* stage, *N* stage, *M* factor, the number of lymph node metastases, *P* stage, lymphatic invasion (ly), blood vessel invasion (v), and the rate of curative resection.

**Fig. 1:**
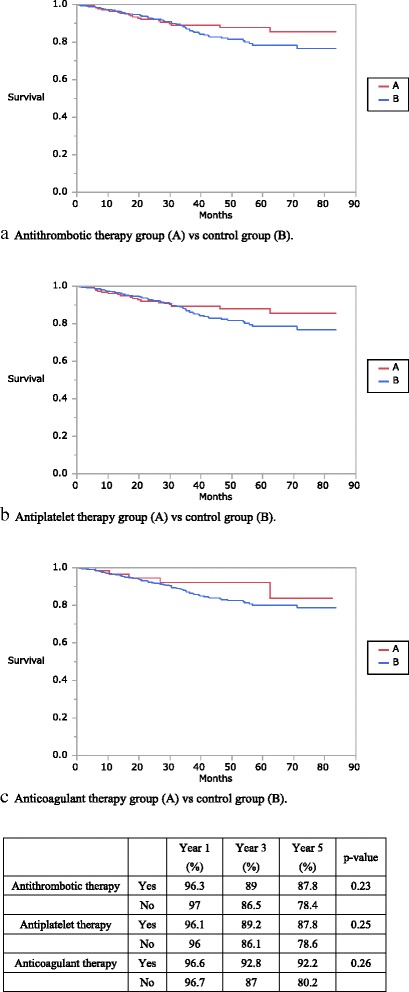
Kaplan–Meier curves showing the overall survival of patients with all-stage colorectal cancer. The antithrombotic, antiplatelet, and anticoagulant therapy groups are compared with the control group

**Fig. 2:**
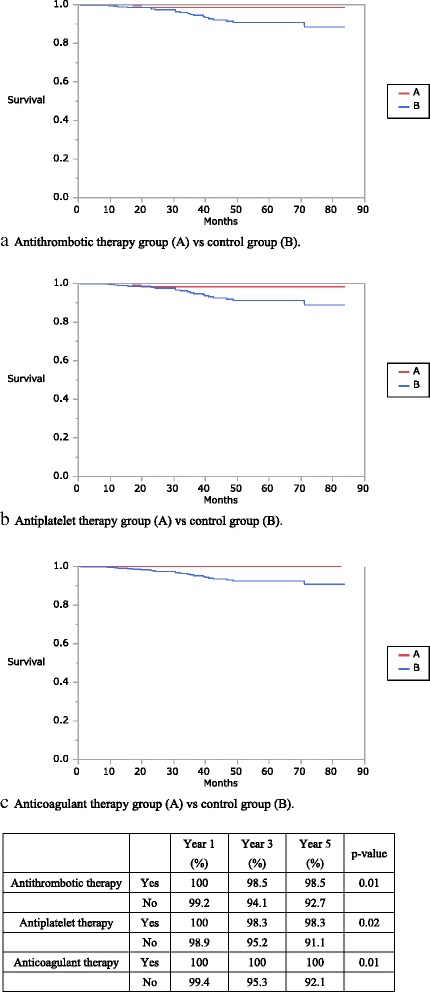
Kaplan–Meier curves of overall survival of patients with stage 0–III colorectal cancer. The antithrombotic, antiplatelet, and anticoagulant therapy groups are compared with the control group

In the survival analysis, long-term ATT, antiplatelet therapy, and anticoagulant therapy affected the prognosis of all-stage and stage 0–III colorectal cancers in the univariate analysis. For all disease stages combined, no benefit was observed for the ATT, antiplatelet therapy, and anticoagulant therapy groups in the OS (ATT: 87.8 vs. 78.4%, *P* = 0.23; antiplatelet therapy: 87.8 vs. 78.6%, *P* = 0.25; and anticoagulant therapy: 92.2 vs. 80.2%, *P* = 0.26; Fig. 1). However, receiving ATT, antiplatelet therapy, or anticoagulant therapy significantly improved OS in patients with stage 0–III colorectal cancer (ATT: 98.5 vs. 92.7%, *P* = 0.01; antiplatelet therapy: 98.3 vs. 91.1%, *P* = 0.02; and anticoagulant therapy: 100 vs. 92.1%, *P* = 0.00; Fig. 2).

## Discussion

The aim of this study was to evaluate the prognosis among patients with colorectal cancer who underwent surgery while receiving ATT. This is the first study to investigate the benefits of ATT in a cohort of Asian patients with colorectal cancer. In this single-center analysis of 710 patients, receiving ATT, antiplatelet therapy, and anticoagulant therapy did not improve OS for patients of all disease stages combined. However, receiving ATT, antiplatelet therapy, or anticoagulant therapy improved OS to a statistically significant level (*P* = 0.01) in patients with stage 0–III colorectal cancer.

We currently do not have enough evidence to prove that the prognosis of cancer is related to the use of antithrombotic agents. Nevertheless, the results for ATT provide proof of principle for pharmacological intervention, such as aspirin, specifically to prevent distant metastasis. Receiving ATT significantly improved OS in stage 0–III colorectal cancer. Previous studies have reported that aspirin was associated with improved survival in stage 0–III colorectal cancer, which suggests that aspirin has a specific effect on the prevention or progression of micro metastases [17, 18]. In the present study, the rate of recurrence was significantly more in the ATT group than that in the control group (14.6 vs. 21.1%, *P* = 0.03, univariate analysis). Although, the mechanisms by which ATT inhibits tumor development remain unclear.

A long prospective study of about 50,000 patients demonstrated that regular, long-term aspirin use reduced the risk of colorectal cancer among men [19]. Recently, a large prospective cohort study showed that aspirin users have a better prognosis in non-advanced colorectal cancer [5]. The Women’s Health Study showed that aspirin has an effect upon a reduction in distant metastasis and improvement in colorectal cancer outcomes [13, 14]. In a meta-analysis of five cardiovascular trials that evaluated 17,285 individuals randomized to daily aspirin user vs non-user, patients with colorectal cancer who received aspirin had the greatest risk of reduction in metastasis (HR = 0.26; 95% CI = 0.11–0.57) [13].

There are plenty of data on aspirin, whereas there is little evidence linking other antiplatelet agents with cancer. Despite clopidogrel being one of the most commonly prescribed drugs in the world, data regarding its effects on cancer are sparse. CHARISMA trial suggested that dual antiplatelet therapy with low-dose aspirin and clopidogrel demonstrated lower new cancer rates than low-dose aspirin only [20]. On the other hand, nitric oxide is associated with increased colorectal cancer risks [21]. A previous report showed that clopidogrel are known nitric oxide inhibitors by suppressing basal and beta-adreno receptor platelet nitric oxide synthase activity [22].

Until now, the mechanisms by which anticoagulant therapy may inhibit tumor development remain unclear. Furthermore, the relationship between anticoagulant therapy and cancer has rarely been reported. A previous report demonstrated that heparin showed multiple actions that may affect the malignancy, especially metastasis [23]. Heparin prevents the formation of tumor-generated thrombin [24]. Conversely, heparin causes tumor angiogenesis inhibiting vascular endothelial growth factor, tissue factor, and platelet-activating factor [25–27].

In contrast, the TRITON-TIMI 38 trial showed the first alarming sign that dual antiplatelet therapy may cause an excess of new cancers as observed with the use of prasugrel [15]. The cancer-associated risks with the long-term use of the previous generation antiplatelet agents such as clopidogrel are not known. The chemical structure of these drugs is very similar to prasugrel. Aggressive platelet inhibition may collapse these benign protective mechanisms, resulting in significantly higher cancer rates [28]. A previous report suggested that antiplatelet therapy and cancer was due to platelet inhibition, promoting easier dissemination of younger unclassified cancer cells, and elevated metastasis risks [29]. Chronic ATT may interrupt the essential abilities of stabilizing tumor cell arrest in the vasculature. If the hypothesis that aggressive platelet inhibition causes higher cancer rates turns out to be true, then chronic antiplatelet therapy should be reconsidered.

This study had some limitations inherent to observational studies that should be addressed. First, we utilized OS and not cancer-specific survival in the evaluation of the prognosis for colorectal cancer. This was because of the greater possibility of cardiac or cerebral events occurring in the patients receiving ATT. In addition, the mean age was also significantly higher in the ATT group than that in the control group. Furthermore, the possibility of death caused by cardiac or cerebral events was higher. This was supported by the higher rate of all-cause mortality in the ATT group, despite no deterioration in OS. In this study, receiving ATT, antiplatelet therapy, and anticoagulant therapy improved OS for patients of stage I–III cancers combined.

Second, no patient in this study received the newer antiplatelet therapy. The TRITON-TIMI 38 trial showed the first alarming indication that dual antiplatelet therapy may cause an excess of new cancers, as observed with prasugrel [15]. In the present study, there was improved OS among all-stage patients who received ATT, although this was not statistically significant. In a future study, we plan to confirm the effects of newer antiplatelet therapy in colorectal cancer.

Finally, this study was a single-center retrospective review with a limited number of patients. However, the patients in the two groups exhibited similar characteristics. To elucidate the risk of dual antiplatelet therapy, there is a need for adequately sized randomized controlled trials designed specifically to look at cancer, especially with newer antiplatelet agents. Furthermore, to identify a molecular mechanism and biomarker related to the heterogeneity of ATT’s effect on tumors, future molecular epidemiologic and experimental studies are needed. A multicenter randomized controlled study that compares ATT and non-ATT patients with colorectal cancer is required to confirm these findings.

## Conclusion

Receiving ATT significantly improved OS in patients with stage 0–III colorectal cancer. However, this study was only the beginning, and multicenter randomized controlled studies are required to determine the effect of ATT on colorectal cancer. In addition, we need to elucidate the mechanisms by which ATT improves prognosis in colorectal cancer.
